# Modification of Initial Highly Active Antiretroviral Therapy (HAART) Regimen in Paediatric HIV Patients

**DOI:** 10.2174/1874613601812010011

**Published:** 2018-03-12

**Authors:** Yee Shan Low, Farida Islahudin, Kamarul Azahar Mohd Razali, Shafnah Adnan

**Affiliations:** 1 Faculty of Pharmacy, Universiti Kebangsaan Malaysia, Jalan Raja Muda Abdul Aziz, 50300 Kuala Lumpur, Malaysia; 2 Hospital Kuala Lumpur, Jalan Pahang, 50586 Kuala Lumpur, Malaysia

**Keywords:** Paediatric, HIV, Regimen modification, Highly active antiretroviral therapy, HAART, Malaysia, Intolerable taste

## Abstract

**Background:**

Treatment options among Human Immunodeficiency Virus (HIV)-infected children are limited as only a few Highly Active Antiretroviral Therapy (HAART) are approved worldwide for paediatric use. Among children, frequent changes in HAART regimen can rapidly exhaust treatment options, and information addressing this issue is scarce.

**Objective:**

The aim of the study was to determine factors associated with the modification of initial HAART regimen modification among HIV-infected children.

**Method:**

A retrospective study was performed among HIV-infected children aged 18 and below, that received HAART for at least six months in a tertiary hospital in Malaysia. Factors associated with modification of initial HAART regimen were investigated.

**Results:**

Out of 99 patients, 71.1% (n=71) required initial HAART regime modification. The most common reason for HAART modification was treatment failure (n=39, 54.9%). Other reasons included drug toxicity (n=14, 19.7%), change to fixed-dose products (n=11, 15.5%), product discontinuation (n=4, 5.6%) and intolerable taste (n=3, 4.2%). The overall mean time retention on initial HAART before regimen modification was 3.32 year ± 2.24 years (95% CI, 2.79–3.85). Patient's adherence was the only factor associated with initial regimen modification in this study. Participants with poor adherence showed a five-fold risk of having their initial HAART regimen modified compared to those with good adherence (adjusted OR [95% CI], 5.250 [1.614 – 17.076], p = 0.006).

**Conclusion:**

Poor adherence was significantly associated with initial regimen modification, intervention to improve patient's adherence is necessary to prevent multiple regimen modification among HIV-infected children.

## 1 INTRODUCTION

Approximately 2.1 million children worldwide are living with Human Immunodeficiency Virus (HIV) in 2016 [1]. This number accounts for about 9% of total people infected with HIV around the world [1]. Unfortunately, an estimated 43% of children infected with HIV have access to antiretroviral treatment [1]. In Malaysia, it is estimated that 92,895 people are living with HIV, with children contributing to approximately 1% of the total number of cases [2].

Although there is no safe and effective cure for human immunodeficiency virus (HIV) infection [3], the use of Highly Active Antiretroviral Therapy (HAART) in the treatment of HIV has substantially reduced the morbidity and mortality associated with HIV infection [3]. Fortunately, the introduction of HAART has revolutionized the management and prognosis of HIV infection in children, making it medically manageable [4]. The increased life expectancy for HIV-infected children in the era of HAART has led to challenges in selecting successive new antiretroviral drug regimens. Given the fact that children may require longer treatment periods, maintaining efficacy is vital. However, despite the advance in drug discovery research and development, there are only a few antiretrovirals approved worldwide for the treatment of HIV infection in children [5, 6]. In developing countries, treatment choice is further restricted by accessibility and cost.

Multiple or frequent regimen modification may rapidly exhaust treatment options among paediatric HIV patients [5, 6]. This is evident as fewer treatment and formulations are available for paediatric HIV patients compared to adults [5, 6]. Poor clinical outcome and the successive regimen are often more complex, less tolerable, and generally inferior to that of first line regimens with a higher risk of treatment failure, and is associated with early HAART modification [7]. Nevertheless, the incidence of initial HAART regimen modification or discontinuation was found to be frequent [8-10]. Reasons reported for initial HAART regimen modification included treatment failure, antiretroviral toxicity, new drug available, cost, suboptimal regimen, and presence of comorbidities [8-10]. Risk factors such as poor patient adherence, development of drug resistance, low baseline CD4 count on HAART initiation, gender, and age have also been attributed to HAART regimen modification [10, 11]. However, most published studies involve adult HIV patients.

There is a lack of data addressing the reasons and factors associated with initial HAART regimen modification among paediatric HIV patients in the Malaysian clinical setting. It is crucial to understand the factors that lead to the initial HAART regimen modification. This is especially true in the paediatric population in order to assist in better decision making of first HAART choice. Optimization of initial HAART in younger patients could lead to better interventions, as well as reduced risk of abrupt HAART interruption. The aim of this study was thus, to determine the factors associated with initial HAART regimen modification among paediatric HIV patients who receive HAART treatment in Malaysia.

## 2 MATERIALS AND METHODS

### 2.1 Study Design

This was a retrospective study, performed in a Malaysian paediatric infectious disease referral centre in Hospital Kuala Lumpur, Kuala Lumpur, Malaysia. HIV-infected children, aged 18 and below, that had received HAART (a combination of three antiretrovirals) for at least 6 months from January 2008 to December 2015, were included. The HAART regimen included in the study were two Nucleoside Reverse Transcriptase Inhibitors (NRTI) - zidovudine and lamivudine, and a Non-Nucleoside Reverse Transcriptase Inhibitors (NNRTI) or Protease Inhibitor (PI).

### 2.2 Data Collection

Patient's information was collected by using a standardized patient data collection sheet. The information included patient's age on HAART initiation, gender, race, main carer during HAART treatment, World Health Organization (WHO) clinical disease staging at HAART initiation, baseline immunodeficiency staging using CD4 count at HAART initiation, baseline viral load, initial HAART regimen used, any regimen modification, reason for modification if occurred, the new HAART regimen used, follow up viral load and immunodeficiency staging using CD4 count at the time of regimen modification, history of previous antiretroviral exposure before HAART initiation and patients’ adherence level to HAART treatment.

### 2.3 Study Definition

In this study, HAART was defined as: a regimen that consists of at least three antiretroviral drugs from at least two drug classes that were approved for HIV infection based on international and national guidelines [11, 12]. Regimen modification was defined as: a switch or substitution of at least one drug from the original HAART regimen [12, 13]. Clinical staging and baseline immunodeficiency staging using CD4 count were classified according to the WHO guidelines [12]. The baseline CD4 counts were divided into four categories: not significant, mild, advanced and severe [12, 13]. CD4-based assessment of immune suppression in children is age-dependent (Table **1**) [12, 13]. Treatment failure was defined as: either clinical failure, immunological failure or virological failure in accordance with WHO definition [13]. Previous antiretroviral exposure was defined as: the use of mono- or dual antiretroviral agent for the purpose of neonatal post-exposure prophylaxis. Adherence to HAART was defined into two categories; good adherence (an adherence pill count/ liquid volume assessment of ≥ 95%, or physician-medical reports of good compliance), and poor adherence (an adherence pill count/ liquid volume assessment < 95%, or physician medical-reports of poor compliance). Pill counts/ liquid volume assessment and adherence counselling is performed by the pharmacist during each clinic session. Physician-medical reports are based on interview-based assessments on whether the patient admitted to non-adherence (missing more than 1 dose) or full adherence since the previous follow-up. The age group of the patient was classified according to the WHO classification to ensure consistency with WHO guidelines in the management of HIV in children [13].

### 2.4 Data Analyses

Data was analysed using SPSS (Statistical Package for the Social Sciences, SPSS Inc., Chicago IL) version 23. Descriptive statistics were used to determine overall frequency of initial HAART regimen modification and to identify the most common reason for regimen modification. Univariate and multivariate logistic regression analysis was conducted to determine factors associated with initial HAART regimen modification. Factors tested in the univariate analysis were gender, ethnicity, age on HAART initiation, main carer, adherence, antiretroviral exposure before HAART, initial HAART regimen, baseline immunodeficiency at HAART initiation and WHO clinical stage at HAART initiation. Crude and adjusted odd ratios (OR), 95% confidence interval (CI) and p-value were presented for regression. The mean duration of retention on initial HAART before regimen modification was analysed using ANOVA or T-test. A p-value ≤ 0.05 was considered significant.

## 3 RESULTS

### 3.1 Demographics

Demographic characteristics of the patients (age, gender, ethnicity, and type of school area) are shown in Table **1**. A total of 110 paediatric HIV infected patient records were screened from January 1, 2008 to December 31, 2015. A total of eleven patients were excluded as HAART was initiated for less than six months. Patient's demographic profiles and clinical characteristic are shown in Table **1**. All patients were diagnosed as mother-to-child transmission patients. Among the 99 patients, 53.5% (n = 53) were male. The ethnicity of more than half of the participants were Malay (n=51, 51.1%), followed by Chinese (n=19, 19.2%), Indian (n=6, 6.1%), indigenous (n=6, 6.1%) and others (n=17, 17.2%). Other ethnicities were patients mainly from Myanmar and Thailand.

### 3.2 Modification of Initial HAART Regimen

The overall percentage of HIV-infected children that required initial HAART regimen modification in this study was 71.1% (n = 71). Modification of two or more agents (treatment failure and drug toxicity) occurred in 53 (52.5%) patients. Treatment failure defined as virological or clinical or immunological failure was the most common reason for initial HAART regimen modification within the studied population (54.9%, n = 39), whilst drug toxicity occurred in 14 (19.7%) patients. Modification of one agent occurred in 18 (17.8%) patients and accounted for the remaining cases that required initial HAART regimen modification: a change to fixed-dose products (n=11, 15.5%), product discontinuation (n=4, 5.6%) and intolerable taste (n=3, 4.2%).

The median duration of retention time on initial HAART before a regimen modification among the study population was 3 years (IQR 2). The time taken for modification of two or more agents was a median of 3.3 years (IQR 2), whilst it took a median of 2.5 years (IQR 0.2) for modification of one agent (p >0.05). Patients with good adherence showed a significantly longer mean retention time on initial HAART (median 4 years, IQR 2.19) compared to patients with poor adherence (median 2.7 years, IQR 1.8) (p = 0.013). No other significant findings were observed.

### 3.3 Factors Associated with Modification of Initial HAART Regimen

In order to assess factors affecting modification in initial HAART regimens, a univariable analysis was performed (Table **2**). It was demonstrated that the factors affecting initial HAART regimen modification were previous antiretroviral exposure (χ^2^ = 5.6, p = 0.018), adherence (χ^2^ = 10.3, p = 0.001), and WHO clinical stage at HAART initiation (χ^2^ = 8.6, p = 0.036). Patients with a previous exposure to antiretroviral were 8.5-fold more likely to modify initial HAART regimen compared to those that were not exposed. Poor adherence increased the risk of modification of initial HAART by 5.8-fold compared to good adherence.

Multivariable logistic regression analysis was conducted to determine the factors associated with initial HAART regimen modification when adjusting for potential confounders (Table **2**). Results showed that only patients' adherence level remained associated with initial HAART regimen modification in the studied population in a logistic multivariate analysis. Patients with poor adherence demonstrated were 5.2-fold more likely to have their initial HAART regimen modified compared to those with good adherence (adjusted OR [95% CI], 5.250 [1.614 – 17.076], p = 0.006).

## 4 DISCUSSION

As in most parts of the world, the younger population in Malaysia remains vulnerable to HIV. In the current work, the ethnic balance of HIV paediatric patients observed, clearly represents the Malaysian population [14]. Free HAART treatment for paediatric HIV patients are currently provided as part of the Ministry of Health, Malaysia initiative in expanding the availability and accessibility of treatment in the country [14].

HAART is vital for maximal clinical benefit in HIV-infected paediatric patients [12]. Despite this, initial HAART regimen modification is found to be common in clinical practice [8-10, 15]. In this current study, results revealed initial HAART regimen modification occurred in the majority of the paediatric HIV patients. Early modification of initial HAART however, has been associated with poor clinical outcome [16]. In instances where modification involves two or more agents as observed in more than half of the study population, the succeeding regimen is always more complex, less tolerable and is associated with a higher risk of treatment failure [7, 16]. Changes of more than one agent usually occurs in treatment failure [7, 16], similarly observed in the current work. In children, studies have reported a 34% treatment failure rate that led to a first-line regimen modification in a five-year follow up [11]. HIV-children in Thailand demonstrated a 33% treatment failure and HAART modification on first-line HAART within 96 weeks of follow up [16]. Similarly, the main reason for initial HAART modification in this current work was treatment failure that occurred within an average of three years. Treatment failure has been accounted for majority of the cases that require HAART regimen modification in both children and adults [17-19]. However, other reasons include drug toxicity, previous antiretroviral exposure, clinical stage and non-adherence [9, 17, 18], similarly observed in the present study.

Various factors have led to initial HAART modification [11]. Among factors that have been identified are poor adherence, antiretroviral drug resistance, poor absorption, exposure to a single dose nevirapine, low baseline CD4 count at HAART initiation and demographic data such as age, gender and main carer [11, 15, 20-22]. In this current work, poor adherence was the single factor that significantly led to modification of initial HAART regimen. The current study further demonstrated that patients with poor adherence were found to be five-times more likely to have their initial HAART regimen modified compared to those with good adherence. Adherence plays a vital role in the treatment of HIV infection [23-27]. Nonetheless, poor adherence continues to be a predominant factor for both HAART modification and treatment failure in both adult and children HIV patients [11, 16, 23-27]. Lack of adherence to HAART results in suboptimal antiretroviral plasma concentration, leading to unsatisfactory viral suppression [16]. In turn, this promotes viral resistance, which eventually leads to treatment failure and regimen modification [16, 25].

Reasons for poor adherence to HAART among HIV infected children is often multifactorial [25, 28, 29]. In general, factors that influence adherence in children can be divided into three main groups, patient or family/carer-related factors, medication-related factors and healthcare delivery system-related factors [30, 31]. About one third of patients in this study were under the care of a children's home, which may be a potential problem for lack of adherence. The current setting in the local children’s home is such that no specific drug management is available for HIV patients. Children living with a non-parent carer have been demonstrated to worse adherence status in paediatric HIV patients [32]. Other reasons for poor adherence in children include psycho-developmental stages [31], increasing age [33], side effect profile, palatability and complexity of the antiretroviral regimen [6, 15, 30, 31]. A better understanding of factors contributing to poor adherence among HIV-infected children is crucial to prevent frequent regimen modification, which can rapidly lead to antiretroviral treatment option exhaustion among this vulnerable group. Nonetheless, the current study was able to identify factors that affected HAART management in children. Addressing issues regarding poor adherence among children with HIV infection is important as the need for long term HAART could ultimately increase the risk of drug resistance.

As with all studies, there were a few limitations in the current work. Firstly, being a retrospective study, the accuracy of the data could not be confirmed with the patients or clinicians. Furthermore, there were only three participants who fell within the age category of 10-19 years old, and a larger, multi-centred study could reduce these limitations in future work. More importantly, it should be noted that this study intended to serve as a baseline data of paediatric HAART modification in the local setting, looking into general changes of HAART. Thus, the broader term of HAART modification was used in the current work, taking into account major modifications involving two or more agents such as drug toxicity, and minor modifications involving one agent such as product discontinuation. However, findings suggest that drug toxicity and product discontinuation may not be related to differences in adherence. Further work could be performed by separating these two outcomes for a more specific results. In addition to this, other variables such as calendar year effects of drug modification may also affect clinical outcome of HAART. Hence, generalizability of the study should be done with caution.

## CONCLUSION

In conclusion, the overall percentage of children infected with HIV that required initial HAART regimen modification was high and the most common reason for initial HAART modification was treatment failure. Poor adherence was found to be an independent predictor of initial HAART regimen modification within the local population. It is important to note that although HIV clinics tend to stress on the importance of adherence to all patients, other factors may also affect medication adherence. Furthermore, findings of the study suggest that more work should be performed to determine factors that may affect HAART modification as adherence of HAART medication may not apply to modifications involving toxicity and product discontinuation.

## Figures and Tables

**Table 1:** Patient demographics and clinical characteristics at HAART initiation.

**Characteristic**	**Initial HAART Modification**		**Total**	**χ^2^ (df)**	**p-value^a^**
	**Yes**	**No**	**n (%)**		
Overall	71	28	99 (100)	–	–
**Sex**	–	–	–	0.0 (1)	0.996
Female	33	13	46 (46.5)	–	–
Male	38	15	53 (53.5)	–	–
**Race**	–	–	–	7.0 (4)	0.136
Malay	39	12	51 (51.5)	–	–
Chinese	15	4	19 (19.2)	–	–
Indian	5	1	6 (6.1)	–	–
Indigenous	5	1	6 (6.1)	–	–
Others	7	10	17 (17.2)	–	–
**Age on HAART Initiation**	–	–	0.2 (2)	0.862
< 1 year	19	9	28 (28.3)	–	–
1 year to < 10 years	50	18	68 (68.7)	–	–
10 years to 19 years	2	1	3 (3.0)	–	–
**Main Carer**	–	–	–	1.2 (3)	0.748
Biological parent	26	12	38 (38.4)	–	–
Adoptive parent	9	2	11 (11.1)	–	–
Relatives	14	4	18 (18.2)	–	–
Children's home	22	10	32 (32.3)	–	–
**Adherence**	–	–	–	10.3 (1)	0.001
Good	36	24	60 (60.6)	–	–
Poor	35	4	39 (39.4)	–	–
**Initial HAART Regimen^b^**	–	–	–	4.5 (2)	0.103
2 NRTI plus NVP	32	19	51 (51.5)	–	–
2 NRTI plus EFV	21	6	27 (27.3)	–	–
2 NRTI plus LR	18	3	21 (21.2)	–	–
**Antiretroviral Exposure Before HAART**	–	–	–	5.6 (1)	0.018
Yes	17	1	18 (18.2)	–	–
No	54	27	81 (81.8)	–	–
**Baseline Immunodeficiency at HAART Initiation^c^**	–	–	–	0.9 (3)	0.830
Not significant	9	2	11 (12.4)	–	–
Mild	10	3	13 (14.6)	–	–
Advanced	13	5	18 (20.2)	–	–
Severe	30	17	47 (52.8)	–	–
**WHO Clinical Stage at HAART Initiation**	–	–	–	4.4 (3)	0.225
Stage I (n=20)	17	3	20 (23)	–	–
Stage II (n=7)	6	1	7 (8.0)	–	–
Stage III (n=31)	23	8	31 (35.6)	–	–
Stage IV (n=29)	16	13	29 (33.3)	–	–

^a^ p-value < 0.05 was considered significant ^b^ NRTI -Nucleoside reverse transcriptase inhibitors, NVP- nevirapine, EFV-efavirenz, LR-lopinavir/ritonavir ^c^ CD4% counts categorized as not significant was defined as CD4% >35% for children less than 11 months or >30% for children 12-35 months or >25% for children 36-59 months or >500 cell/mm^3^ for children 5 years and older. CD4% counts categorized as mild was defined as CD4 30-35% for children less than 11 months or CD4 25-30% for children 12-35 months or 20-25% for children 36-59 months or CD4 350-499 cell/mm^3^ for children 5 years and older. CD4% counts categorized as advanced was defined as CD4 25-29% for children less than 11 months or CD4 20-24% for children 12-35 months or CD4 15-19% for children 36-59 months or CD4 200-349 cell/mm^3^ for children 5 years and older. CD4% counts categorized as severe was defined as CD4 <25% for children less than 11 months or CD4 <20% for children 12-35 months or CD4 <15% for children 36- 59 months or CD4 <200cell/mm^3^ for children 5 years and older [12, 13].

**Table 2:** Logistic regression analysis describing correlates of patient's demographic and clinical characteristic with occurrence of initial HAART regimen modification among HIV-infected children.

**Variables**	**Logistic univariate analysis**			**Logistic multivariate analysis**		
	**Crude OR**	**95% CI**	**p-value^a^**	**Adjusted OR**	**95% CI**	**p-value^a^**
**Sex**	–	–	–	–	–	–
Female	1.000	–	–	–	–	–
Male	0.998	0.415-2.399	0.996	–	–	–
**Race**	–	–	–	–	–	–
Malay	1.000	–	–	–	–	–
Chinese	1.154	0.321-4.145	0.826	–	–	–
Indian	1.538	0.163-14.486	0.707	–	–	–
Indigenous	1.538	0.163-14.486	0.707	–	–	–
Others	0.215	0.067-0.689	0.010	–	–	–
**Age on HAART initiation**	–	–	–	–	–	–
< 1 year	1.000	–	–	–	–	–
1 year to < 10 years	1.316	0.504-3.432	0.575	–	–	–
10 years to 19 years	0.947	0.076-11.870	0.967	–	–	–
**Main caretaker**	–	–	–	–	–	–
Biological parent	1.000	–	–	–	–	–
Adoptive parent	2.077	0.388-11.121	0.393	–	–	–
Relatives	1.615	0.438-5.956	0.471	–	–	–
Children's home	1.015	0.369-2.797	0.976	–	–	–
**Adherence^b^**	–	–	–	–	–	–
Good	1.000	–	–	–	–	–
Poor	5.833	1.836-18.538	0.003	5.250	1.614-17.076	0.006
**Antiretroviral exposure before HAART^c^**	–	–	–	–	–	–
No	1.000	–	–	–	–	–
Yes	8.500	1.074-67.295	0.043	–	–	–
**Initial HAART regimen^d^**	–	–	–	–	–	–
2 NRTI plus NVP	1.000	–	–	–	–	–
2 NRTI plus EFV	2.078	0.713-6.060	0.180	–	–	–
2 NRTI plus PI	3.562	0.926-13.710	0.065	–	–	–
**Baseline immunodeficiency at HAART initiation^e^**	–	–	–	–	–	–
Not significant	1.000	–	–	–	–	–
Mild	0.741	0.100-5.490	0.769	–	–	–
Advanced	0.578	0.091-3.663	0.560	–	–	–
Severe	0.392	0.076-2.029	0.264	–	–	–
**WHO clinical stage at HAART initiation**	–	–	–	–	–	–
Stage I (n=20)	1.000	–	–	–	–	–
Stage II (n=7)	1.059	0.092-12.234	0.963	–	–	–
Stage III (n=31)	0.507	0.117-2.201	0.365	–	–	–
Stage IV (n=29)	0.217	0.052-0.907	0.036	–	–	–

^a^ p-value < 0.05 was considered significant ^b^ NRTI -Nucleoside reverse transcriptase inhibitors, NVP- nevirapine, EFV-efavirenz, LR-lopinavir/ritonavir ^c^ CD4% counts categorized as not significant was defined as CD4% >35% for children less than 11 months or >30% for children 12-35 months or >25% for children 36-59 months or >500 cell/mm^3^ for children 5 years and older. CD4% counts categorized as mild was defined as CD4 30-35% for children less than 11 months or CD4 25-30% for children 12-35 months or 20-25% for children 36-59 months or CD4 350-499 cell/mm^3^ for children 5 years and older. CD4% counts categorized as advanced was defined as CD4 25-29% for children less than 11 months or CD4 20-24% for children 12-35 months or CD4 15-19% for children 36-59 months or CD4 200-349 cell/mm^3^ for children 5 years and older. CD4% counts categorized as severe was defined as CD4 <25% for children less than 11 months or CD4 <20% for children 12-35 months or CD4 <15% for children 36- 59 months or CD4 <200cell/mm^3^ for children 5 years and older [12, 13].
